# Lassa fever presenting as acute abdomen: a case series

**DOI:** 10.1186/1743-422X-10-123

**Published:** 2013-04-19

**Authors:** Andrew E Dongo, Emeka B Kesieme, Christopher E Iyamu, Peter O Okokhere, Odigie C Akhuemokhan, George O Akpede

**Affiliations:** 1 Department of Surgery, Irrua Specialist Teaching Hospital, PMB 08, Irrua, Edo State, Nigeria; 2 Department of Internal Medicine, Irrua Specialist Teaching Hospital, Irrua, Edo State, Nigeria; 3 Department of Paediatrics, Irrua Specialist Teaching Hospital, Irrua, Edo State, Nigeria

**Keywords:** Lassa fever diagnosis, West African surgeon, Acute abdomen

## Abstract

Lassa fever, an endemic zoonotic viral infection in West Africa, presents with varied symptoms including fever, vomiting, retrosternal pain, abdominal pain, sore-throat, mucosal bleeding, seizures and coma. When fever and abdominal pain are the main presenting symptoms, and a diagnosis of acute abdomen is entertained, Lassa fever is rarely considered in the differential diagnosis, even in endemic areas. Rather the diagnosis of Lassa fever is suspected only after surgical intervention. Therefore, such patients often undergo unnecessary surgery with resultant delay in the commencement of ribavirin therapy. This increases morbidity and mortality and the risk of nosocomial transmission to hospital staff.

We report 7 patients aged between 17 months and 40 years who had operative intervention for suspected appendicitis, perforated typhoid ileitis, intussuception and ruptured ectopic pregnancy after routine investigations. All seven were post-operatively confirmed as Lassa fever cases. Four patients died postoperatively, most before commencement of ribavirin, while the other three patients eventually recovered with appropriate antibiotic treatment including intravenous ribavirin.

Surgeons working in West Africa should include Lassa fever in the differential diagnosis of acute abdomen, especially appendicitis. The presence of high grade fever, proteinuria and thrombocytopenia in patients with acute abdomen should heighten the suspicion of Lassa fever. Prolonged intra-operative bleeding should not only raise suspicion of the disease but also serve to initiate precautions to prevent nosocomial transmission.

## Background

Exploratory laparotomies are carried out for patients in emergency situations when there is little time left for any exhaustive investigations in the face of deterioration in a patient’s clinical condition. They are often carried out after active resuscitation and preliminary investigations [1]. However, there are some situations when exploratory laparotomies actually worsen outcome and endanger the lives of the operators and other health care workers.

Surgical intervention in Lassa fever is hazardous in the extreme. Lassa fever is a viral haemorrhagic fever with a high case fatality rate of between 20–58% [2]. When recognized, treatment is with ribavirin, [3,4] an antiviral agent, and supportive care. Intravenous administration of ribavirin is known to reduce mortality from about 55% to 5% [4]. The symptoms of Lassa fever are varied and non- specific. The clinical diagnosis is difficult in the early course of the disease [5,6]. Even when suspected, the confirmatory laboratory diagnosis is not within the confines of many centres in the West African sub-region where Lassa fever is endemic [6]. In recent times, however, due to collaborative efforts, laboratory confirmation of clinically suspected cases of Lassa fever using ELISA and RT-PCR techniques is now possible in some countries in West Africa [7,8]. Progress in infrastructural development, technology and capacity building has made it possible to make rapid diagnosis of Lassa fever, using point-of contact rapid diagnostic test (RDT), and also permit the comprehensive monitoring of life-threatening cases of Lassa fever, principally due to the ability to measure and characterize immunologic and metabolic parameters in some centers in West Africa [8].

This disease may in fact be easily confused with many other tropical illnesses like perforated typhoid ileitis [2,5,9] with which it shares many epidemiologic variables like poor hygiene, poor refuse disposal and overcrowded living conditions. It is therefore pertinent for all surgeons in areas, known to be endemic for Lassa fever, to have a high index of suspicion when reviewing suspected cases of acute abdomen with fever and when urinalysis shows protein or blood. Meticulous attention to universal precautions is important to prevent nosocomial transmission.

**Figure 1:**
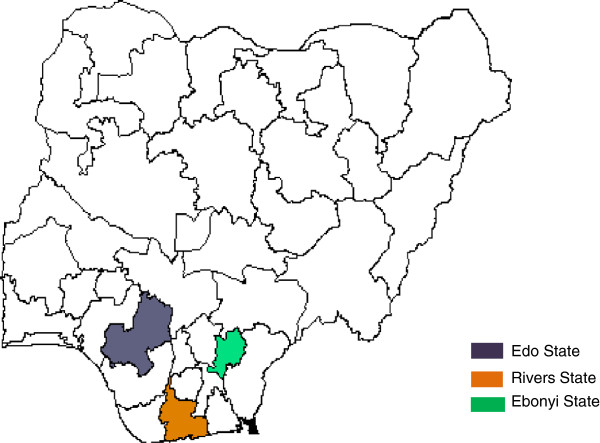
Map of Nigeria showing States where cases of acute abdomen from Lassa fever were reported.

We report seven cases of patients with Lassa fever seen between 2009 and 2012, who had inadvertent surgical intervention in Nigeria for presumed surgical causes. All seven had confirmatory laboratory diagnosis post operatively by Reverse Transcriptase Polymerase chain reaction (RT-PCR) technique at the Nigerian Government Institute for Lassa Fever Research and Control at Irrua, Edo State, Nigeria. Three of these cases had their surgery at Irrua Specialist Teaching Hospital, Edo State. Two had surgery at private hospitals near Irrua in Edo State. One had surgery in Port Harcourt, Rivers State, where Lassa fever activity was not previously reported. The last had surgery at Abakaliki, Ebonyi State (Figure 1). Approval was received from the Ethics and Research committee of Irrua Specialist Teaching Hospital, Irrua, before the study was undertaken.

### Case 1

A 28-year old undergraduate of Lagos State University presented to Irrua Specialist Teaching Hospital, Irrua, Nigeria in January 2009 with a 2 week history of fever and abdominal pain. He had self medicated with anti-malarial drugs. When he did not get better, he visited a private hospital in Lagos, Nigeria, and was admitted for 1 week and treated for malaria. He had antibiotics also for suspected typhoid fever. When he did not improve, he requested for discharge and came back home to Ekpoma in Edo State. High grade fever, with chills and rigors, continued. The abdominal pain which was initially periumbilical, soon became generalized. He developed 2-3 episodes daily of non-projectile vomiting for 2 days prior to presentation at Irrua Specialist Teaching Hospital.

At presentation, he was found to be acutely ill looking and dehydrated with a temperature of 38.9°C. He had generalized abdominal tenderness which was worse in the right iliac fossa. He also had rebound tenderness and guarding. Bowel sounds were normoactive. A diagnosis of perforated appendicitis and a differential diagnosis of perforated typhoid ileitis were made. The investigations done at presentation (see Table 1) included packed cell volume (PCV) which was 34%, white blood cell count was (WBC) 2,700/mm^3^ with lymphocytosis of 84%, ESR was 90 mm/hr; the serum urea was elevated (77 mg/dl). At laparotomy, we found hyperaemia involving the appendix and terminal ileum, ascites of about 500 mls, multiple enlarged mesenteric lymph nodes and multiple pale patches in the anti-mesenteric border of the ileum. He had appendicectomy and lavage of peritoneum. His clinical course deteriorated over the next 72 hours after surgery with persisting pyrexia and respiratory difficulty. Persistent pyrexia which was non-responsive to antimalarials and antibiotics post surgery in a Lassa fever endemic area led to the suspicion of Lassa fever. It was subsequently confirmed with Lassa virus R-T PCR test done on the 3^rd^ post operative day (POD). He was commenced on a 10 day course of intravenous ribavirin on the 4^th^ day post surgery, and made a dramatic and uneventful recovery. The dose of ribavirin was 100 mg/kg in day 1, followed by 25 mg/kg daily (days 2–6) and 12.5 mg/kg daily (days 7–10). This regimen is adapted from Davidson Principles and Practice of Medicine [10]. He was discharged 2 weeks after surgery.

**Table 1:** Temperature and clinical laboratory parameters on admission, surgical observations and outcome in 7 patients with Lassa fever and acute surgical abdomen

**Case**	**Temp on admission °C**	**Liver enzymes**		**Blood count**		**Urinalysis**		**Bleeding at surgery**	**Outcome**
		**AST**	**ALT**	**WBC**	**Platelet**	**Protein**	**Blood**		
1	38.9°C	N/D	N/D	2700	N/D	N/D	N/D	Normal	Recovered
2	39°C	35	25	3700	229,000	3+	2+	Normal	Recovered
3	39°C	N/D	N/D	N/D	N/D	N/D	N/D	prolonged	Died
4	38.1°C	N/D	N/D	N/D	N/D	N/D	N/D	prolonged	Died
5	38.3°C	N/D	N/D	N/D	N/D	1+	N/D	prolonged	Died
6	39.2°C	N/D	N/D	19500	N/D	1+	1+	prolonged	Died
7	39^.^2°C	38	16	7000	34,000	Nil	2+	Normal	Recovered

*WBC* White blood cell count.

WBC: normal range 3.4-9.06 × 10^9^/L.

Platelet count: normal range 165-415 × 10^9^/L.

AST/ALT: normal range 12-38 IU/L.

Bleeding time: normal <7.1 min.

### Case 2

A 27 year old woman had appendicectomy in a private clinic at Ubiaja, Edo State, for suspected appendicitis. She however continued to experience fever, malaise and headache after surgery. There was no history of any patient at the private clinic having clinical features suggestive of Lassa fever while she was there. She was referred to Irrua Specialist Teaching Hospital after 7 days, in February 2010, for further management.

At presentation, the temperature was 39°C. She had generalized abdominal tenderness on examination. An Abdominopelvic ultrasound scan done showed a small collection around the appendiceal stump. Being from an area where Lassa fever activity has been reported coupled with the persistent fever, Lassa fever was suspected and confirmed by laboratory test. PCV at presentation was 28%, with a WBC of 3,700/mm^3^ and neutrophil and lymphocyte counts of 30% and 43% respectively. ESR was 120 mm/hr. Platelet count was 229,000/mm^3^; AST was 35 IU/L, ALT was 25 IU/L; urinalysis showed protein (3+) and blood (2+). She made an uneventful recovery after a 10 day course of ribavirin treatment which was started 8 days after surgery, but the 2nd day after presentation to Irrua Specialist Teaching Hospital.

### Case 3

A 16 year old male was admitted via the emergency room of Irrua Specialist Teaching Hospital in August 2010 after appendicectomy in a private hospital in Ekpoma a few hours earlier. He was noticed to have prolonged bleeding during surgery. His clinical state did not improve after surgery and he remained febrile and pale. He was transfused with one unit of whole blood. He was referred on the first post operative day. At presentation, he was toxic looking and febrile. Temperature was 39°C. He died within 24 hours of admission before a confirmatory laboratory test result for Lassa virus was received. He did not receive ribavirin. The surgeon who carried out the operation developed a febrile illness after 16 days with prominent cervical and axillary lymphadenopathy. He was subsequently confirmed to have Lassa fever as well. He made uneventful recovery after a 10 day course of ribavirin.

### Case 4

A 17 month old male child was brought into the Children’s Emergency Room of Irrua Specialist Teaching Hospital in October 2010, with complaints of abdominal pain of 6 days, fever of 3 days and 2 days of passage of bloody stools. He had received blood transfusion, intravenous fluids, anti-malarials and antibiotics at the referral hospital before being verbally referred.

On examination he was acutely ill and pale. His temperature was 38.1°C. He had prolonged bleeding from needle puncture sites. He was however haemodynamically stable. An abdominal examination showed a cylindrical mass in the left iliac fossa which was 4 cm in its widest diameter and hepatosplenomegaly. There was a red fleshy mass protruding from the anus with a soft mass palpable in the rectum on digital rectal examination. A diagnosis of colocolic intussusceptions was made and he was taken for emergency laparotomy. Lassa fever was suspected, based on prolonged bleeding from puncture sites, a sign which raises the suspicion of Lassa fever in an endemic area.

Operation findings were those of a hypertrophied, recto-sigmoid segment with clots of blood in the lumen but no lead point or telescoping of the gut. A tissue biopsy of the sigmoid loop was sent for histology and it showed a normal biopsy. The test result received post-operatively was positive for Lassa fever. Despite the prompt commencement of intravenous ribavirin and serial fresh whole blood transfusions, he died after 21 hours on admission.

### Case 5

A 25 year old woman with amenorrhoea of 10 weeks duration, presented to the University Teaching Hospital at Abakaliki, Ebonyi State, South East Nigeria. She had fever and rigors for one week and had not responded to anti-malarials and antibiotics. She developed abdominal pains for 2 days before presentation. At presentation, her axillary temperature was 38.3°C. A pregnancy test was positive. Urinalysis showed proteinuria (1+) and casts (2+). A positive tap of non-clotting blood led to a diagnosis of ruptured ectopic gestation. She had exploratory laparotomy at which no ectopic pregnancy was found. A suspected inflamed appendix was removed by the general surgeon who was invited. However, she continued to bleed from the wound edges. She was re-explored and was discovered to be bleeding intraperitonealy. The abnormal bleeding led to suspicion of Lassa fever and a blood sample was sent to Irrua for confirmatory diagnosis. She later died in the intensive care unit before the result was received. She was not treated with ribavirin. Two gynaecologists, two surgeons, an anaesthetist and a nurse anaesthetist took ill with Lassa fever between 6-9 days later. They did not receive post exposure prophylaxis. They were all transferred to Irrua Specialist Teaching Hospital for treatment. One death was recorded.

### Case 6

A 40 year old man presented to a private hospital in Port Harcourt, Rivers State, Nigeria in March 2012 with a 3 day history of abdominal pain which shifted from the epigastrium to the right iliac fossa. He vomited twice on the day of admission.

At presentation his axillary temperature was 39.2°C. He was pale. The pulse rate was 116/minute. He had tenderness in the right iliac fossa with rebound. PCV was 28%, WBC was 19,500/mm^3^ with a neutrophil count of 58% and lymphocyte of 41%; urinalysis showed Protein(+), Blood(+) Epithelial cells (+) and Casts(+). An ultrasound scan suggested an inflamed appendix. Other findings were normal. He had appendicectomy. Findings at surgery included a slightly inflamed appendix and prolonged oozing of blood from the mesoappendix.

About 8 hours after surgery, he was noticed to be very pale with hypotension. He consequently had an exploratory laparotomy and about 2 litres of non-clotting blood was evacuated from the peritoneal cavity. Oozing was noted from the undersurface of the liver, appendiceal stump and mesoappendix. Haemostasis was attempted with reinforced sutures. About 700 mls of blood was recorded from a drainage bag about 4 hours after surgery. He was transfused with 1 pint of whole blood intraoperatively and six pints postoperatively. He continued to have pyrexia and died after referral to the teaching hospital in Port Harcourt, on the 3rd post-operative day (POD). He was not treated with ribavirin. A blood sample tested positive for Lassa virus. The surgeon took ill 11 days later with fever and neurological symptoms. Lassa fever was suspected because he had managed a fatal case of Lassa fever a few weeks earlier, and because he did not respond to anti-malarial and antibiotics. He tested positive for Lassa virus and was transferred to Irrua Specialist Teaching Hospital, Irrua, Nigeria for treatment. He was treated with intravenous ribavirin and made an uneventful recovery.

### Case 7

A 24 year old undergraduate was referred from the University Health Centre Ekpoma to Irrua Specialist Teaching Hospital with a diagnosis of acute appendicitis in March 2012. She had been ill for 5 days with fever, chills and rigors. She developed lower abdominal pain 2 days before presentation. She had two episodes of non projectile vomiting on the day of presentation. There was no urinary symptom. On examination, she was acutely ill looking. Her axillary temperature was 39.2°C. The abdomen moved with respiration. She had marked tenderness with rebound in the right iliac fossa. Vaginal and rectal examinations were normal. PCV was 34%, total WBC was 7000/mm^3^, with neutrophil of 72% and lymphocyte of 24%; ESR was 105 mm/hr; AST was 38 IU/L, ALT was 16 IU/L; urinalysis showed blood(2+); platelet count was 34,000/mm^3^; serum urea was 22 mg/dl. An abdominopelvic ultrasound scan was essentially normal.

She had emergency appendicectomy. The intraoperative finding was hyperaemia of the serosa of the vermiform appendix. Post operatively, she continued to have daily spikes of temperature of between 38°C and 39°C. Urine and blood cultures were normal. She was treated for scanty malaria parasitaemia with arthemeter injections. On the 5th post-operative day, because of persistent fever which did not respond to the use of antimalarial and antibiotics, and being in an endemic area, Lassa fever was suspected; the test was positive. She made an uneventful recovery after treatment with a 10 day course of parenteral ribavirin therapy, starting on the 6th post-operative day. Histology of the appendix specimen revealed evidence of tissue edema and neutrophil infiltration in the muscularis propria.

## Discussion

Lassa fever is one of the major causes of febrile illnesses in West Africa [11]. It is however often unrecognized [9]. The reasons for this include a general lack of awareness amongst medical practitioners in the sub-region, the varied and non specific clinical features of the early disease and the inability to carry out confirmatory laboratory diagnosis in many centres in the sub-region.

In Lassa fever infection, gastrointestinal symptoms, including vomiting and abdominal pain have been found to be fairly common [6,7,11]. In a study from Irrua, Nigeria, abdominal pain was the 4th most common presenting symptom [7], occurring in about 36% of patients. A higher rate of just under 50% has been previously described [11]. The abdominal pain in Lassa fever has been described as either localized or generalized. Two of our patients (cases 6 and 7) had localized abdominal pain in the right iliac fossa with tenderness and rebound tenderness, while one had initial localized abdominal pain which became generalized. Four patients had diffuse abdominal pain. The presence of localized right iliac fossa pain and tenderness led to a diagnosis of acute appendicitis and subsequent surgical intervention.

Histological examination of two of these patients showed evidence of neutrophil infiltration in the muscularis propria implying that there was an inflammatory process involving the appendix. Acute appendicitis is a known differential diagnosis of arenavirus infection [6], although, many surgeons who diagnose appendicitis will hardly consider this etiology. Such localized peritonitis may be due to an active viral replication in the lymphoid tissue of the appendix [12].

Diffuse abdominal pain described as cramping pain associated with diarrhoea and vomiting [11], is known also to occur in Lassa fever. But we now describe generalized abdominal tenderness with rebound tenderness in a patient. This patient had ascites at exploratory laparotomy (case 1). We suggest that the abdominal pain in some Lassa fever patients with acute abdomen may be due to peritoneal inflammation. Late onset of poyserositis has been associated with Lassa fever with effusion in the various serous cavities [13]. We now report a case of ascites occurring in the acute phase of Lassa fever infection. It is likely that Lassa virus antigen, elaborated during the course of Lassa fever illness, may produce an abnormal inflammatory response in mesothelial surfaces that might result in the effusion seen in Lassa fever [12].

The second case of generalized abdominal tenderness was intriguing. The triad of abdominal pain, left iliac fossa mass and bleeding per-rectum suggests intussuception. Intussuception involving the colon is known to be common in certain parts of Africa [14]. A viral adenitis on the other hand can provoke intussuception. This patient had prolonged bleeding from needle puncture sites pre-operatively, yet, neither the pediatricians who first reviewed nor surgeons who were later invited was willing to delay intervention for the eight hours needed for a diagnostic sample for Lassa virus to be processed. The strong clinical diagnosis of intussuception outweighed any other considerations. At time of surgery however, only an intraluminal clot was found. The presence of thickened and edematous colonic wall suggested possibly a spontaneous reduction. However, it is also likely that the clot was what simulated an intussuceptum when the preoperative rectal examination was done.

Four of our cases had evidence of intraoperative bleeding disorder. Bleeding in Lassa fever has been reported as often only mucosal [3,5], and Lassa fever has sometimes been dismissed as pathologically not a haemorrhagic fever [15]. Nevertheless, different surgeons in the index reports described prolonged bleeding from cut surfaces. The cases from Port Harcourt, Rivers State and Abakaliki, Ebonyi State demonstrated massive post operative bleeding which required re-operation and blood transfusion. Abnormal bleeding from puncture sites and wound edges as illustrated by cases 3, 4, and 6 should be a pointer to the likelihood of Lassa fever in febrile patients with acute abdomen.

Attending surgeons involved in three of the four cases with bleeding developed Lassa fever. Nosocomial human to human transmission has been described previously [6] and surgeons in Nigeria have been known to succumb to Lassa fever in the past after treating infected patients [16,17]. None of the surgeons involved had a needle stick injury.

We now also describe two anaesthetists who developed Lassa fever infection after anaethesizing Case 5 with one mortality. They reported no needle stick injury or contact with body fluids while the surgical operation was going on. Direct aerolisation from the process of intubation and ventilation may be a plausible explanation for the infection.

Two of the index cases (5 and 6) contracted the disease in areas not previously known to report cases of Lassa fever. The other 5 cases were resident in areas known to report cases of the disease. However, there was no indication that the health facilities where they initially presented had cases with fever associated with abnormal bleeding suggestive of Lassa fever.

The definitive treatment of Lassa fever is with ribavirin, but proper supportive measures can also positively influence outcome. Although ribavirin has been shown to reduce mortality significantly when started within 6 days of illness, it also has varying degrees of effectiveness in the later stages of Lassa fever [4]. Parenteral administration is considered more effective than oral preparations [4]. Barrier nursing in an isolation ward is mandatory to reduce nosocomial spread. The use of personal protective equipment by those who provide care and the practice of universal precaution are important measures that must be adhered to by all concerned to reduce human to human spread of the disease.

Post exposure prophylaxis (PEP) is recommended for definite high risk exposure [6], for instance, for those who have had penetrating injuries with contaminated sharp instruments, mucosal surfaces and cuts contamination with infected blood and body fluids of Lassa fever patients and medical personnel involved in resuscitation or intubation of seriously ill Lassa fever patients [18]. There exists a direct relationship between the intensity of viral load and severity of Lassa fever illness [4]. In view of the foregoing, and exemplified by the occurrence of Lassa fever in two of the medical staff (anaesthetists) in Abakaliki who had no direct contact with the patient’s blood or body fluids during surgery, it can be argued that PEP may be beneficial to theatre personnel who manage patients with severe Lassa fever even if there is no direct contact with blood or body secretions because of the possibility of aerosol spread facilitated by the enclosed nature of the theatre. There is no prospective clinical study on the use of ribavirin or any other antiviral drug in PEP. A retrospective study in Sierra Leone on the use of ribavirin for PEP showed variations in ribavirin doses, frequency of dosing and duration of PEP [19]. The proposed guidelines for ribaivrin use for PEP by Bausch *et al*[18] are likely to be beneficial. However, when fever develops during the period of PEP, full treatment using standard treatment regimen should be started.

This case series highlights the additional hazards practitioners in the West African sub-region are exposed to. Unfortunately, the exact burden of such risks would remain unknown for the foreseeable future until Lassa fever diagnosis can be made readily in more central reference laboratories or rapid bed side diagnostic kits become available in peripheral health facilities in endemic areas.

In our review of these cases, we have attempted to identify predictors of possible Lassa fever infection for surgeons who do not have ready access to PCR diagnosis. Table 1 shows some of the findings in the work up of these patients. Proteinuria, leucopenia and thrombocytopenia are known to be laboratory features of acute Lassa fever infection in many patients. These were present in some our patients. Proteinuria is an important component of the clinical predictors for the diagnosis of Lassa fever [11], and it becomes a feature from 4 to 7 days of the course (stage 2) of the disease [20].

## Conclusion

We suggest that in patients with acute abdomen presenting with high fever, proteinuria or hematuria and thrombocytopenia should raise suspicion of the possibility of Lassa fever.

The presence of extensive bleeding intra-operatively, and/or oozing of blood from suture sites post-operatively, in a patient with acute abdomen presenting with fever makes the likelihood of Lassa fever even higher, and may be a pointer to severity and possible risk for nosocomial transmission to attending health care providers.

### Consent

Written informed consents were obtained from the patients and relatives for publication of these Case series. Copies of the written consents are available for review by the Editor-in-Chief of this journal

## Abbreviations

R-T PCR: Reverse Transcriptase Polymerase chain reaction; PCV: Packed cell volume; WBC: White blood cell count; POD: Postoperative day; PCV: Packed cell volume; WBC: White blood cell count; POD: Postoperative day.

## Competing interests

The authors declare that they have no competing interests

## Authors’ contributions

AED conceived of the study, helped in coordination, contributed in drafting and revision of the manuscript. EBK helped in coordination and drafting of the manuscript. CEI contributed in collection of data and drafting of the manuscript. POO contributed in drafting and revision of the manuscript. OCA contributed in data collection and drafting of the manuscript. GOA contributed in design of the study and drafting of the manuscript. All authors read and approved the final manuscript.

## Authors’ information

Apart from CEI, who is a Senior Medical Officer, the other coauthors are Consultant staff at the Irrua Specialist Teaching Hospital in Irrua, Nigeria.
